# Innovation networks in the advanced medical equipment industry: supporting regional digital health systems from a local–national perspective

**DOI:** 10.3389/fpubh.2025.1635475

**Published:** 2025-07-31

**Authors:** Feng Hu, Huijie Yang, Liping Qiu, Xiaoping Wang, Zhimin Ren, Shaobin Wei, Haiyan Zhou, Yufeng Chen, Hao Hu

**Affiliations:** ^1^Institute of International Business & Economics Innovation and Governance, Shanghai University of International Business and Economics, Shanghai, China; ^2^International Business School, Shanghai University of International Business and Economics, Shanghai, China; ^3^CEEC Economic and Trade Cooperation Institute, Ningbo University, Ningbo, China; ^4^College of Business Administration, Ningbo University of Finance and Economics, Ningbo, China; ^5^School of Management, Zhejiang Gongshang University Hangzhou College of Commerce, Hangzhou, China; ^6^Institute of Digital Economy and Financial Powerhouse Building, Guangdong University of Finance, Guangzhou, China; ^7^Graduate School, Nueva Ecija University of Science and Technology, Cabanatuan, Philippines; ^8^School of Economics and Management, Zhejiang Normal University, Jinhua, China; ^9^School of Economics, Shanghai University, Shanghai, China

**Keywords:** medical equipment, innovation network, spatial pattern, core cities, public health

## Abstract

This study investigates the spatial structure and influencing factors of the innovation network in the advanced medical equipment and device manufacturing industry in the Yangtze River Delta (YRD). This industry is crucial to public health system resilience and regional industrial upgrading. Drawing on joint patent data from the IncoPat Global Patent Database covering the period 2005–2024, the study constructs innovation networks and conducts comparative analyses from both national and regional perspectives using social network analysis and geodetector methods. The findings reveal that: (1) At the national level, the innovation network has gradually expanded but remains sparse, characterized by a “core–periphery” structure dominated by Beijing and Shanghai. Urban participation in central and western China is limited. Short-term objectives often drive industry–university–research collaborations, and collaborations in basic research among academic institutions remain insufficient. (2) In contrast, the YRD region has formed a denser, polycentric network, with Shanghai as the primary hub and Nanjing and Suzhou as key secondary centers. Local enterprises exhibit strong incentives to engage in collaborative innovation within industry–university–research frameworks. (3) Geodetector analyses indicate that economic development, technological capability, and government policy support are major drivers of network formation, while infrastructure quality plays a crucial enabling role. The relative importance of these factors varies between national and regional levels. These results provide empirical evidence to inform regional innovation strategies and targeted policy design for strengthening collaboration in the advanced medical equipment industry.

## 1 Introduction

The advanced medical equipment and device manufacturing industry is a strategic emerging industry in China. Its development meets the growing demand for high-quality medical services and drives the transformation and upgrading of the national economy while improving China’s technological capabilities and innovation capacity in the medical sector (1–5). In 2023, the total revenue of China’s medical device industry reached 1.31 trillion yuan with a compound annual growth rate of 10%, making it the second-largest market globally.^1^ However, more than 80% of advanced medical equipment, such as radiotherapy equipment and MRI machines, continues to rely on imports.^2^ The Yangtze River Delta (YRD), which is endowed with abundant innovation resources and a highly skilled talent pool, emphasizes the development of advanced medical equipment and device manufacturing. As a core agglomeration area for China’s medical device industry, the YRD hosts more than 20% of the nation’s clinical and animal testing institutions for medical devices, with universities that offer medical device-related programs leading the country.^3^ Additionally, the region pioneered the Medical Device Marketing Authorization Holder system, which lays a solid foundation for the high-quality, integrated development of the industry.

Innovation serves as a crucial driver of high-quality regional economic development. The COVID-19 pandemic highlighted the vulnerabilities in global medical supply chains and emphasized the need for domestically driven innovation and production capacity. In this context, developing a resilient and localized medical innovation ecosystem is crucial for addressing future public health crises. The flow of innovation elements among cities that form innovation networks has drawn considerable attention from scholars and has become a central theme in research on innovation geography (6–10). In constructing innovation networks, studies have utilized data sources such as patent collaborations, transfers, citations, academic paper coauthorships, research project collaborations, and high-end talent mobility. These studies have applied social network analysis (SNA) methods to examine innovation networks (11–15). Early research primarily adopted a micro-level perspective, focusing on innovation actors such as enterprises, research institutions, and government organizations, and examined the structure of innovation networks as well as the interactions among these entities (16–22). As research has deepened, particularly in the knowledge economy era with the relational turn in economic geography and the widespread influence of network science, scholars have increasingly shifted to macro-level studies that investigate innovation networks at the national, urban cluster, and provincial levels. This shift has placed greater emphasis on the structural characteristics, influencing factors, and evolutionary processes of innovation networks (23–32). For instance, Feng Hu et al. identified a global scale-free innovation network centered around Europe and North America using cooperative patent data (33), while Yang et al. examined collaborative innovation in China’s telecommunications sector through negative binomial regression (34). Other researchers have analyzed innovation networks in regional contexts such as Anhui Province or across national urban systems. These studies confirm the importance of network centrality, connection strength, and knowledge flow efficiency in shaping innovation outcomes (35–38).

However, relatively few studies have specifically examined the advanced medical equipment industry in the Yangtze River Delta (YRD)—a key cluster region of China’s advanced manufacturing and public health technology innovation. Zhang and Rao conducted a macro-level SWOT–ANP analysis of China’s advanced medical equipment industry, highlighting systemic challenges such as weak enterprise R&D capabilities and fragmented university–industry cooperation (39). While their study offers important strategic insights, it lacks a spatial-network perspective and does not focus on regional collaboration patterns. Similarly, Lai et al. evaluated the innovation efficiency of the pharmaceutical manufacturing industry from a technology gap perspective, revealing regional disparities but without addressing inter-organizational innovation networks or patent-based collaboration structures (40).

Existing literature tends to focus on policy strategy, industrial risk, or innovation efficiency measurement at a provincial level, but seldom integrates relational data (e.g., joint patent applications) to analyze how innovation actors connect and form networks within a region. The present study addresses this gap by constructing a dynamic innovation network for the advanced medical equipment industry in the YRD, using longitudinal patent co-application data and comparative analysis across national and regional scales. This approach enables us to uncover how economic, technological, and institutional factors jointly shape collaborative structures in the advanced medical equipment and device manufacturing industry.

However, current cross-scale urban innovation network studies suffer from methodological limitations and cognitive gaps, and a shift beyond single spatial dimensions is required. The integration of the “global–local” (glocalization) paradigm is necessary to analyze the complex spatial characteristics of innovation cooperation (41–47). Furthermore, previous studies have focused primarily on innovation networks at the urban level with limited attention to heterogeneous characteristics across specific industries. Previous studies also lack sufficient consideration of how industry attributes influence network structures.

From an industrial perspective, studies of the advanced medical equipment and device manufacturing industry have focused mainly on its industrial development (2, 48–51), spatial distribution (5, 52, 53), trade patterns, and competitiveness (54–57). While these studies have provided in-depth analyses of the spatial–temporal characteristics of the industry, they often overlook relational perspectives. There is an urgent need to integrate network embeddedness effects and endogenous-exogenous driving mechanisms to explore innovation pathways within the framework of flow-space restructuring. Recent findings also suggest a significant positive spatial correlation between regional public health development and the efficiency of collaborative medical innovation, underscoring the importance of understanding spatial linkages in industry-specific innovation networks (58).

Given this context, this study examines the innovation network of the advanced medical equipment and device manufacturing industry and analyses the structural characteristics of the regional innovation network in the YRD from both National-Regional Perspectives. This approach facilitates the identification of innovation development patterns in the industry, promotes the optimal allocation of resources, and accelerates the localization of advanced medical equipment and devices in preparation for potential public health crises. Additionally, it provides theoretical foundations and practical references for national policy formulation and coordinated innovation development in the YRD. This study is important in two key ways. First, this study leverages nearly two decades of phased data and employs social network analysis to address the limitations of traditional regional economic studies in dynamic network topology analysis, thereby enriching the theoretical framework of the evolution of innovation networks. Second, through the construction of a dual-perspective (“national-regional”) analytical framework and comparative analysis of cross-scale innovation network indicators, this study uncovers the integration and flow characteristics of innovation elements at different spatial scales. This approach surpasses the limitations of single-scale research, offers a novel stratified paradigm for studying industrial innovation networks, and provides practical insights for optimizing innovation-based responses during health emergencies, enhancing urban resilience against future shocks.

## 2 Research data and methods

### 2.1 Research subjects and data

Based on the classification codes of strategic emerging industries combined with the national economic industry codes, patents related to the advanced medical equipment and device manufacturing industry over the past two decades (2005–2024) were retrieved from the Incopat Global Patent Database. Only patents with at least two applicants in the patent ownership claims were considered, resulting in a total of 35,094 relevant patents. When patent collaborations were calculated, if a joint application for a patent was made by applicants A, B, and C, collaborations were counted as one instance each for AB, AC, and so forth. Data retrieval was conducted on February 10, 2025.

To objectively capture the evolutionary characteristics of the innovation network in the advanced medical equipment and device manufacturing industry, a time-segmented research approach was adopted. The study period was divided into four time intervals: 2005–2009, 2010–2014, 2015–2019, and 2020–2024.

### 2.2 Research methods

#### 2.2.1 Social network analysis (SNA)

This study applies social network analysis (SNA) to examine the structural characteristics of innovation collaboration networks in the advanced medical equipment and device manufacturing industry. Based on joint patent data from 2005 to 2024, innovation networks are constructed where nodes represent firms, universities, or research institutions, and edges represent co-application relationships. The networks are directed and weighted, with edge weights corresponding to the number of joint patent applications. Key metrics such as network density, modularity, degree centrality, average path length, and clustering coefficient are used to assess the scale, clustering, and actor roles in the network. National and Yangtze River Delta (YRD) regional networks are analyzed separately to capture both macro and localized collaboration dynamics. This method helps reveal the evolution and spatial heterogeneity of innovation linkages in a strategically significant industry (59–64).

#### 2.2.2 Geodetector analysis

The factor detection module of the geodetector method was used to analyze the relationship among the weighted centrality of the innovation network and influencing factors such as urban economic development, technological innovation, the policy/social environment, and infrastructure from a National-Regional Perspective (65–68).

## 3 Evolution of the innovation network in the advanced medical equipment and device manufacturing industry from a national-regional perspective

### 3.1 Structural characteristics of the innovation network in the YRD from a national perspective

The number of participating cities and network edges in the innovation network increased from 72 and 129 in Phase I to 271 and 2,073 in Phase IV (Table 1), respectively. However, the overall network density remained low, indicating that from a national perspective, there is still considerable room for collaborative innovation within the YRD’s advanced medical equipment and device manufacturing industry. The network modularity index initially increased and then decreased; the average clustering coefficient increased continuously, and the average path length decreased continuously. These patterns suggest that regional network connections have become more tightly knit and that the community structure has become increasingly complex.

**Table 1:** Statistical characteristics of the innovation network from a national perspective.

Statistical characteristic	Phase I	Phase II	Phase III	Phase IV
Number of nodes	72	126	212	271
Number of edges	129	316	834	2,073
Network density	0.025	0.020	0.019	0.028
Modularity index	0.393	0.402	0.375	0.290
Average clustering coefficient	0.139	0.253	0.258	0.416
Average path length	2.851	2.760	2.895	2.635

The modularity index reflects the tightness of the network’s community structure. Innovation in advanced medical equipment and devices requires interdisciplinary collaboration across fields such as biomedical engineering and materials science, leading to cross-domain and cross-community characteristics in collaborative networks. Consequently, the modularity index remained relatively high. However, the pilot implementation of the Medical Device Marketing Authorization Holder system in the YRD in 2017 partially weakened the community boundaries defined by institutional types (e.g., Industry-university collaborations), leading to a temporary decline in the modularity index.

From a national perspective, the spatial differentiation of the weighted degree in the overall innovation network of the YRD’s advanced medical equipment and device manufacturing industry is significant. Over the past 20 years, the coefficient of variation for city centrality and weighted centrality in the network has increased from 2.4237 and 1.6177 in the first phase to 3.1924 and 1.7263 in the fourth phase, respectively, indicating that the spatial differentiation of weighted centrality has become more pronounced and that the polarization trend in network connection strength has intensified. Within the YRD, cities such as Shanghai, Suzhou, Nanjing, and Hangzhou, as well as cities outside the region, including Beijing, Shenzhen, and Guangzhou, have become increasingly important in the network. The proportion of cities where the average degree is greater than the average centrality decreased from 20.08% in Phase I to 16.24% in the fourth stage, indicating an increase in network polarization and an increase in the control power of core cities. This suggests a widening gap in innovation levels within the advanced medical equipment and device manufacturing industry for cities in the YRD.

**Figure 1:**
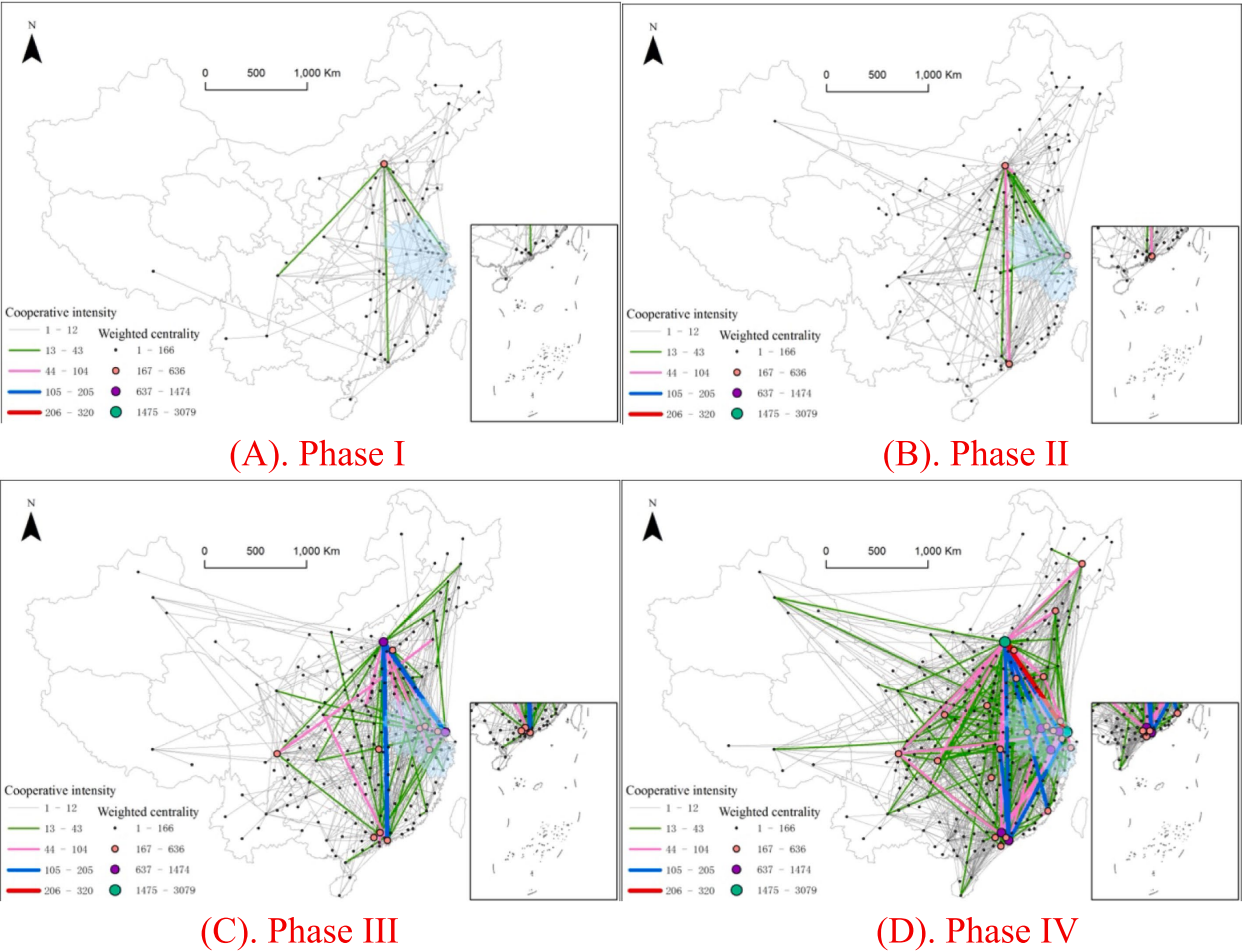
The YRD innovation network from a national perspective over the past 20 years.

Using the natural breaks method in ArcGIS, the cooperative innovation intensity in the fourth phase was classified into five levels and applied to other stages (Figure 1). The intensity and number of innovation collaborations between cities gradually increased. The number of cities in the first and second levels was relatively small, and these levels emerged only in the third and fourth stages. And a very clear distribution of lines is characterized by the fact that the lines are mainly concentrated in the eastern, central, and southern regions, especially the more coastal, the denser the lines. The hierarchical structure of the network changed significantly and formed a spatial pattern with Beijing and Shanghai at the core, radiating nationwide. The cooperation intensity among cities within the YRD and neighboring cities was greater than that among cities in other regions of the country. In Phases I and II, there were no cooperative innovation pairs at the first and second levels. In the third stage, there were no first-level cooperative innovation pairs, and the only second-level pairs were Beijing–Shanghai (192) and Shanghai–Suzhou (106). The third-level pairs included Beijing–Shenzhen, Nanjing–Suzhou, and 18 other pairs. In the fourth stage, there were four first-level cooperative innovation pairs: Shanghai–Suzhou (320), Beijing–Tianjin (312), Nanjing–Zhenjiang (245), and Beijing–Shanghai (235). The second level included 10 pairs, such as Beijing–Shenzhen, Beijing–Suzhou, and Shenzhen–Wuhan. The third level consisted of 34 pairs, including Beijing–Chengdu, Ningbo–Shanghai, and Beijing–Jinan.

The analysis of the top 10 cities in terms of centrality and net inflow within the YRD’s overall innovation network for the advanced medical equipment and device manufacturing industry from a national perspective shows several trends (Appendix Table 1). Within the YRD’s city, Shanghai consistently ranked second in centrality after Beijing, but its net inflow remained negative throughout all phases. Nanjing, Suzhou, and Hangzhou experienced significant increases in net inflow during Phases III and IV. Notably, Nanjing and Hangzhou transitioned from negative net inflow in Phases I and II to positive net inflow in Phases III and IV. This suggests that the two cities are gradually shifting from being exporters to importers of innovation resources and can attract more external innovation resources. The average net inflow remained negative from Phases I to III but became positive in Phase IV with an increasing standard deviation. This trend suggests a significant increase in regional collaboration within the YRD’s innovation network. The network evolved into a structure where Shanghai serves as a supercore city that drives and radiates innovation cooperation across surrounding cities, with highly active collaboration among internal cities.

The dominance of Shanghai can be attributed to its strong research foundation supported by top universities such as Fudan University and Shanghai Jiao Tong University, both of which are in the top 1% of the world in medical-related disciplines according to ESI, coupled with the clinical translation capabilities of institutions such as Shanghai Shende Wuchuang Era Medical Technology and Shanghai Changzheng Hospital. According to the “China Scientific and Technological Achievement Transformation 2024 Annual Report,” Shanghai ranks first in the amount of contracts for transferring, licensing, and investing in the local transformation of universities and institutes (69). These factors have established Shanghai as a core hub for collaborative innovation among industry, university, and research.

Outside the YRD, the influence of cities in the innovation cooperation network is relatively dispersed. Beijing has consistently ranked first in centrality and maintained a substantial lead over other cities, and its net inflow has increased continuously. This dominance is due primarily to Beijing’s strong foundation in basic research, which is supported by leading universities such as Peking University and Tsinghua University, in addition to the industrialization capabilities of companies such as Beijing Natong Medical Technology. Additionally, Beijing benefits from top-tier clinical validation resources provided by institutions such as the Chinese Academy of Medical Sciences’ Peking Union Medical College Hospital and the People’s Liberation Army General Hospital. These factors have fostered a triadic “university-enterprise-hospital” innovation ecosystem. Furthermore, Guangzhou and Shenzhen experienced significant increases in centrality by Phase IV, reaching 141 and 152, respectively, which indicates an expansion in the breadth of innovation cooperation. However, they still lagged behind Beijing, which had a centrality of 224.

Among the cities in central and western China, Chengdu and Wuhan exhibited large fluctuations in net inflow, highlighting the relative independence of most non-YRD cities in the national innovation network. Xi’an and Hefei were only in the top 10 in the first phase and were subsequently overtaken by cities in other geographies. Of the remaining Midwestern cities, only Zhengzhou tied with Qingdao in the second phase. Beijing’s leading role in innovation cooperation continues to strengthen, competition between Guangzhou and Shenzhen remains intense, and cities in central and western China display insufficient stability in innovation collaboration.

For the types of industry-university-research collaboration between the YRD and the rest of the country (Table 2), industry–industry collaboration has shown continuous growth, rising from 13.33% in Phase I to 53.87% in Phase IV and maintaining the leading position since Phase II. Industry–research collaboration peaked at 28.95% in Phase II but gradually declined from Phase III onwards, reaching only 23.42% in Phase IV. Moreover, industry-university collaboration, research-research collaboration, and university-research collaboration have continuously declined, whereas university-university collaboration has fluctuated, with the type of collaboration with the lowest share.

**Table 2:** Types of industry-university-research collaboration in the innovation network from a national perspective.

Collaboration type	Phase I	Phase II	Phase III	Phase IV
Industry–Industry	13.33%	43.09%	50.38%	53.87%
Industry–University	18.10%	14.14%	12.48%	8.86%
Industry–Research	25.71%	28.95%	24.30%	23.42%
University–University	0.95%	2.30%	2.16%	1.26%
University–Research	13.33%	8.22%	5.82%	6.75%
Research–Research	28.57%	3.29%	4.88%	5.83%

Overall, driven by the market demand in the advanced medical equipment and device manufacturing industry, the need for collaborative innovation among enterprises has surged, which has increased the dominance of industry–industry collaboration. This type of collaboration plays a significant role in innovation cooperation between the YRD and the rest of the country. In contrast, knowledge-intensive collaborations such as industry-university and industry-research partnerships have gradually declined. The collaboration between the YRD and the rest of the country tends to be oriented toward short-term benefits, with research–research collaboration experiencing the most rapid decline. This situation highlights the need for improved coordination in basic research.

### 3.2 Structural characteristics of the innovation network in the YRD from a local perspective

**Figure 2:**
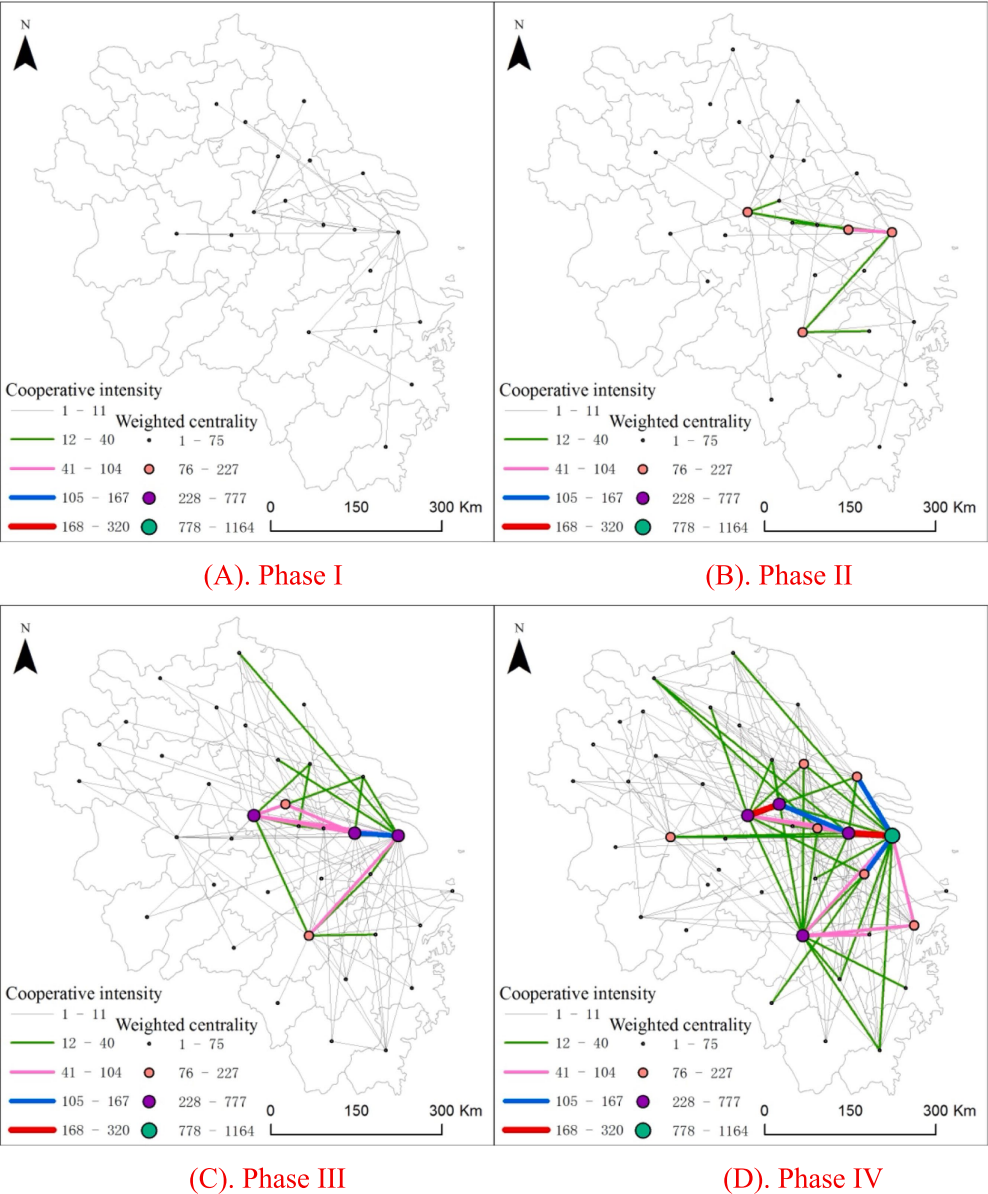
Characteristics of the innovation network structure within the YRD region from a local perspective over the past 20 years.

Figure 2 shows that, from a local perspective, the intensity of collaborative innovation among cities within the YRD in the advanced medical equipment and device manufacturing industry has grown continuously, with increasingly close connections. Consistent with the national trend, the second level occurs in Phase III and the first level in Phase IV, and shows a radiation from the core city to other cities. Moreover, the first and second levels are mostly found among close neighboring cities, and the lower levels can radiate farther away. The number of collaborating cities and network edges increased from 19 cities and 25 edges in Phase I to 40 cities (excluding Huangshan) and 248 edges in Phase IV, covering nearly all cities within the YRD (Table 3). The increasing network density and average clustering coefficient indicate tighter network connections, whereas the declining network modularity index suggests an increase in cross-community collaborative innovation within the network. This trend also reflects a weakening of administrative segmentation between provinces in the region. In contrast, administrative boundaries between provinces are more clearly defined from the national perspective.

**Table 3:** Statistical characteristics of the innovation network from a local perspective.

Statistical indicator	Phase I	Phase II	Phase III	Phase IV
Number of nodes	19	26	36	40
Number of edges	25	58	131	248
Network density	0.073	0.089	0.104	0.159
Modularity index	0.462	0.345	0.264	0.235
Average clustering coefficient	0.103	0.312	0.493	0.566
Average path length	2.261	2.574	2.266	2.099

From a local perspective, the spatial differentiation of weighted degree centrality among cities in the YRD innovation network for the advanced medical equipment and device manufacturing industry is evident. Over the past 20 years, the coefficient of variation for city centrality in the network decreased from 1.0477 in Phase I to 0.9417 in Phase IV, whereas the coefficient of variation for weighted city centrality increased from 1.2007 in Phase I to 1.8240 in Phase IV. This indicates that differences in the breadth of connections among YRD cities have diminished, with peripheral cities increasing their participation in the innovation network. However, disparities in the intensity of collaborative innovation among cities have widened to form a distinct core-periphery structure. The spatial pattern of the YRD innovation network is characterized by Shanghai as the core with Suzhou, Nanjing, Zhenjiang, and Hangzhou as secondary cores, while other cities remain on the periphery.

Using the natural breaks classification method in ArcGIS, the collaborative innovation intensity in Phase IV was divided into five levels and applied to the previous phases. The results show that in Phases I and II, no collaborative innovation pairs were classified at the first or second level. In Phase III, no pairs were at the first level, and only one pair—Shanghai–Suzhou—fell into the second level, with an innovation intensity of 106. In Phase IV, two pairs reached the first level, with Shanghai–Suzhou advancing from the second to the first level and Zhenjiang–Nanjing newly entering the first level. Moreover, three pairs—Suzhou–Zhenjiang, Jiaxing–Shanghai, and Nantong–Shanghai—were classified at the second level.

Overall, collaborative innovation was relatively sparse in the early phases, but by Phases III and IV, network connections had become denser. However, high-level collaborative innovation remained concentrated in the eastern part of the YRD and occurred primarily in core cities such as Shanghai, Suzhou, Nanjing, and Zhenjiang. Furthermore, high-level innovation collaboration was mostly interprovincial; cross-provincial innovation cooperation gradually increased, indicating a weakening of administrative segmentation between provinces.

By analyzing the top 10 cities in terms of centrality and their net inflow degree within the YRD innovation network for the advanced medical equipment and device manufacturing industry from a local perspective (Appendix Table 2), it becomes evident that as the innovation network has continued to develop, Shanghai has gradually become the sole innovation leader in the region, whereas Nanjing has emerged as a key city for innovation collaboration. Hefei is the city with the largest change in centrality, dropping out of the top 10 in the next phase, from sixth in Phase I. The number of cities with a negative net inflow degree decreased from six in Phase I to just one in Phase IV. By Phase IV, only Shanghai had a negative net inflow degree that continued to decline, whereas all other cities had positive values. This trend shows that these YRD cities have absorbed more innovation resources from regional networks than exported, while Shanghai has continued to channel resources to its neighbors, playing the role of a core hub.

Shanghai consistently ranked first in centrality. Its net inflow degree dropped from −15 to −138, reflecting its absolute dominance as the central hub within the innovation network. Nanjing’s rise was particularly notable: its centrality increased from 7 to 42, and its net inflow degree surged from −3 to 240. Since Phase III, Nanjing has become a major innovation collaboration city within the YRD, likely due to its abundant higher education resources and strong local policy support.

Additionally, mid-sized cities such as Nantong and Jiaxing have experienced rapid growth and have gradually transitioned from peripheral cities to secondary core cities. These cities serve as crucial supplements to the collaborative innovation network. Overall, the YRD network exhibits a trend of multicentre coordination and dynamic evolution and forms a three-tiered structure with Shanghai as the core, secondary hubs that provide support, and midsized cities that serve as supplementary nodes.

From a local perspective, with regard to the types of industry-university-research collaboration among cities within the YRD (Table 4), industry–industry collaboration has continuously increased; it rose from 44.23% in Phase I to 66.86% in Phase IV and maintained its leading position throughout. Although industry-university collaboration peaked at 26.96% in Phase II, it declined to 10.82% in Phase IV. Industry–research collaboration and research–research collaboration have fluctuated within the YRD, whereas university–university and university–research collaboration have declined significantly.

**Table 4:** Types of industry-university-research collaboration in the innovation network from a local perspective.

Collaboration type	Phase I	Phase II	Phase III	Phase IV
Industry–Industry	44.23%	51.54%	62.54%	66.86%
Industry–University	21.15%	26.96%	13.05%	10.82%
Industry–Research	15.38%	11.26%	14.40%	11.37%
University–University	7.69%	1.02%	0.79%	1.21%
University–Research	7.69%	3.75%	5.74%	5.59%
Research–Research	3.85%	5.46%	3.49%	4.14%

Compared to the national level, both industry–industry and industry-university collaboration within the YRD increased slightly. The strong demand for collaborative innovation among enterprises highlights the vitality of industry partnerships, and the development of university alliances has yielded positive results. However, university–industry collaboration and university-university collaboration lack a long-term incentive mechanism. In the field of research coordination, while industry-research and research-research collaborations in the YRD demonstrate some resilience compared with the national level, knowledge flow remains weak, possibly due to institutional and systemic barriers.

## 4 Analysis of network influencing factors

### 4.1 Selection of influencing factors

Research on the factors that influence collaborative innovation suggests that a city’s economic development, technological capabilities, infrastructure, and policies significantly affect its participation in collaborative innovation networks (33–42). On the basis of these insights and in line with the principles of comprehensiveness and data availability, the weighted degree centrality of cities in the advanced medical equipment and device manufacturing industry innovation network during the fourth phase is selected as the dependent variable. The independent variables include factors that represent economic development (economic development level, financial support, market activity, industrial economic scale, trade openness, and economic modernization level), technological innovation (technological commercialization capacity, innovation output, and the density of core innovation entities in cities), policy/social factors (talent cultivation support, scientific and technological innovation support, and development zone policies), and infrastructure (telecommunications infrastructure and medical resource levels).

For higher education institutions, double first-class universities are assigned a value of 2, whereas regular higher education institutions are assigned a value of 1. Similarly, for development zones, national-level development zones are assigned a value of 2, whereas provincial-level development zones are assigned a value of 1 to reflect the differences in institutional support, resource allocation, and policy strength between zones of different administrative levels (63). Before geographical detection analysis is conducted, ArcGIS is used to classify the data into five levels using the natural breaks method. The data for the influencing factors were sourced from the China Urban Statistical Yearbook and relevant provincial statistical yearbooks.

### 4.2 Analysis of influencing factor results

The results of the geographical detector analysis (Appendix Table 3) indicate that economic development, technological innovation, policy/social dimensions, and infrastructure all significantly impact the innovation network of the advanced medical equipment and device manufacturing industry from both the national and local perspectives. Factors with a correlation coefficient above 0.8 are classified as core influencing factors.

From a national perspective, financial support, trade openness, technological innovation output, and scientific and technological innovation support are identified as core influencing factors. From a local perspective, financial support, market activity, and telecommunications infrastructure are the core influencing factors.

Economic development is the core driving force of the innovation network of the advanced medical equipment and device manufacturing industry. The economic development level, industrial economic scale, and economic modernization all have significant effects. Financial support exceeds 0.8 from both national and local perspectives, highlighting the critical role of financial institutions in supporting the innovation network. This underscores the strong support provided by capital mobility to industry innovation. Market activity is more influential locally than at the national level, suggesting that consumer demand within the YRD region plays a more prominent role in shaping the innovation network. Additionally, trade openness exhibits a significant disparity between national and local perspectives, with the national level showing a stronger driving force for industrial innovation. This may be due to the varying degrees of export-oriented economic structures within the YRD, which leads to a more dispersed influence at the regional level.

Technological innovation serves as a crucial engine for the innovation networks of the advanced medical equipment and device manufacturing industry. Technological innovation output is a core factor at the national level, which reflects the importance of technology reserves. A vibrant technology transaction market accelerates the flow of innovation elements. Locally, the technological commercialization capability is close to the core threshold, indicating that the YRD relies on an active technology transaction market to drive innovation. However, the overall density of core innovation entities in cities remains low, particularly in the YRD. This may be due to the uneven distribution of high-tech enterprises (e.g., Hangzhou and Suzhou have over 15,000 national-level high-tech enterprises, whereas Zhoushan, Huainan, and Chizhou have only a few hundred), which restricts regional collaboration.

Policy and social factors provide critical support for the innovation network. Scientific and technological innovation support is the only core factor at the national level, which demonstrates the direct impact of government funding on the innovation network. Locally, development zone policies and university resources are close to the core threshold, indicating that the YRD relies on industrial parks and university intellectual resources to drive innovation. Higher education expenditures help to cultivate high-tech innovation talent, which provides research and development personnel for innovative enterprises and enhances their competitive advantage.

Infrastructure serves as the foundational guarantee for the innovation network. Telecommunications infrastructure and medical resources are essential aspects as they attract high-end talent and reduce innovation costs.

## 5 Conclusions and implications

### 5.1 Conclusion

Based on patent data from 2005 to 2014, this study analyses the spatial structure and influencing factors of the innovation network for the advanced medical equipment and device manufacturing industry in the YRD from both national and local perspectives. The following conclusions are drawn.

1. **Network structure characteristics**: At the national level, the innovation network exhibits a polarization pattern that features a strong core and a weak periphery, whereas the YRD forms a multilevel collaborative network with reduced administrative segmentation between provinces.
2. **Imbalance in industry-university-research collaboration**: Industry collaboration dominates the innovation network, but knowledge-intensive cooperation continues to weaken, and basic research collaboration remains insufficient. This imbalance of integration limits the industry’s ability to generate and disseminate critical innovations quickly during emergencies.
3. **Significant regional disparities**: Core cities within the YRD exhibit a high concentration of innovation resources, whereas central and peripheral cities face challenges in maintaining stability, leading to a widening innovation gap. However, mid-sized cities such as Nantong and Jiaxing are gradually evolving into secondary innovation hubs.
4. **Influence factors**: Economic development is the core driving force of the innovation network, technological innovation serves as a crucial engine, policy/social dimensions provide essential support, and infrastructure forms the fundamental guarantee. These factors provide concrete directional advice to policymakers on enhancing public health resilience in the context of crisis prevention.

### 5.2 Implications and recommendations

1. Based on the analysis of influencing factors, government financial support is found to have a direct impact on regional innovation performance. To further strengthen mechanisms for collaborative innovation, policy instruments such as tax incentives and refined benefit-sharing arrangements could be introduced to promote the sustainable development of regional public health industries, especially for medium-sized regional centers (e.g., Nantong, Jiaxing). Technology spillover from core cities such as Beijing and Shanghai should be encouraged, cross-regional coordination should be enhanced, and administrative barriers should be reduced to increase participation from cities in central and western regions.
2. Both nationally and in the YRD, collaboration between universities and research institutions remains limited, particularly in the field of fundamental research. To address this gap, the research evaluation system should be improved by establishing dedicated funding for foundational research activities in emerging innovation regions. In mature academic clusters (e.g., Shanghai, Nanjing, Hangzhou), dual-employment programs between universities and enterprises, as well as joint industry–university–research, could be promoted to increase the depth and frequency of knowledge-intensive collaborations, such as university–university, university–research, and research–research partnerships. These will foster deeper and broader innovation collaborations, improving the capacity to address future challenges.
3. Given that active technology markets play a crucial role in accelerating the flow of innovation resources, it is necessary to promote patent commercialization through well-developed transfer platforms and efficient transaction services in developed innovation centers. Cultivating sub-innovation hubs is conducive to enhancing the robustness of the overall network, which requires strengthening the transformation of scientific and technological achievements, reinforcing the development of supporting infrastructure, and increasing investment in key innovation drivers.

### 5.3 Theoretical contributions

1. This study employs social network analysis to examine the evolving structure of innovation networks and addresses a gap in traditional regional economic research, which often lacks dynamic analysis of network topology. This study contributes to the theoretical framework of the evolution of innovation networks (11–37).
2. By comparing innovation network indicators across different spatial scales, this study reveals how innovation factors integrate and flow at varying levels, which overcomes the limitations of single-scale research. This study introduces a new multilayered approach to research on industrial innovation networks (37–46).
3. This study expands the understanding of the medical device and manufacturing industry and enriches the understanding of industrial organization and industry chain aspects through the perspective of innovation and cooperation, and provides a network location and technology upgrading difficulty embedded perspective to the theory of industrial technology upgrading (2, 5, 48–57).

### 5.4 Limitations and future research directions

1. This study focuses solely on the YRD. Future research could compare findings with other regions, such as the Pearl River Delta and Beijing-Tianjin-Hebei, to extract more universally applicable patterns of the evolution of regional industrial innovation.
2. This study relies on patent collaboration data to analyze the advanced medical equipment and device manufacturing industry. Future studies could integrate multi-source data, e.g., academic collaboration, researcher mobility, project partnerships, and project funding relationships, to provide a more comprehensive and multi-layered understanding of the industry’s innovation landscape.
3. The analysis of influencing factors is based on macroeconomic data and lacks insights into the micro-level behaviors of innovation actors such as enterprises and universities. Future research could employ interviews or textual analysis of collaborative patents and academic papers to explore the underlying mechanisms of innovation networks.
4. The discussion of the global–local paradigm in this study remains largely theoretical. Future research could develop quantitative models by incorporating cross-national patent collaboration data to further explore the intensity and pathways of multiscale innovation factor flows.

## Data Availability

The original contributions presented in the study are included in the article/Supplementary material, further inquiries can be directed to the corresponding authors.

## Footnotes

^1^National Medical Products Administration Southern Medicine Economic Research Institute: *2024 China Medical Device Industry Development Report*, published in November 2024. Available on the official website of the Shanghai Municipal Commission of Economy and Informatization (https://www.sheitc.sh.gov.cn/gydt/20241230/b070b36f40104e2baf8b3caff5e42153.html) [Accessed: March 5, 2025].

^2^Roland Berger: *Current Situation and Trends in China’s Medical Device Industry*, initially published in February 2023 and updated in March 2024 (with supplementary data). Available on the Roland Berger official website (https://www.sgpjbg.com/baogao/116111.html) [Accessed: March 5, 2025].

^3^National Medical Products Administration Southern Medicine Economic Research Institute & NMPA Medical Device Technology Review and Inspection YRD Subcentre: *YRD Medical Device Industry Development Research Report*, jointly published in November 2024. Available at (https://finance.sina.com.cn/money/smjj/2024-11-19/doc-incxhkcn8227431.shtml) [Accessed: March 5, 2025].
