# Supplementary material for: Innovation networks in the advanced medical equipment industry: supporting regional digital health systems from a local–national perspective
Source: Front Public Health. 2025 Jul 31;13:1635475. doi: 10.3389/fpubh.2025.1635475 (PMC12350417; doi:10.3389/fpubh.2025.1635475)
Supplement: Supplementary file 1 [file Supplementary_file_1.docx]

**Appendix Table 1** Top 10 Cities by Centrality in the Innovation Network from a National Perspective

| Rank | **Phase I** | | | **Phase II** | | | **Phase III** | | | **Phase IV** | | |
| --- | --- | --- | --- | --- | --- | --- | --- | --- | --- | --- | --- | --- |
|  | City | Centrality | NID | City | Centrality | NID | City | Centrality | NID | City | Centrality | NID |
| 1 | Beijing | 39 | -17（78/95） | Beijing | 91 | -144（162/306） | Beijing | 146 | 118（750/632） | Beijing | 224 | -417（1331/1748） |
| 2 | Shanghai | 28 | -42（24/66） | Shanghai | 47 | -51（100/151） | Shanghai | 82 | -76（325/401） | Shanghai | 164 | -90（1102/1192） |
| 3 | Shenzhen | 13 | 7（26/19） | Nanjing | 32 | -60（50/110） | Guangzhou | 68 | -93（165/258） | Shenzheng | 141 | 152（813/661） |
| 4 | Nanjing | 11 | -8（5/13） | Shenzhen | 30 | 81（132/51） | Nanjing | 62 | 90（285/195） | Guangzhou | 125 | -111（450/561） |
| 5 | Suzhou | 11 | 8（17/9） | Guangzhou | 26 | -2（55/57） | Shenzhen | 56 | -15（226/241） | Hangzhou | 101 | 136（473/337） |
| 6 | Chengdu | 9 | 15（23/8） | Hangzhou | 20 | -29（40/69） | Suzhou | 43 | -85（181/266） | Suzhou | 101 | 43（723/680） |
| 7 | Guangzhou | 9 | -11（5/16） | Suzhou | 16 | 30（69/39） | Hangzhou | 39 | 13（120/107） | Nanjing | 99 | 239（591/352） |
| 8 | Hangzhou | 7 | -4（5/9） | Tianjin | 16 | 0（60/60） | Wuhan | 38 | 91（162/71） | Chengdu | 89 | 72（317/245） |
| 9 | Hefei | 6 | 5（12/7） | Chengdu | 15 | -5（21/26） | Chengdu | 34 | -97（65/162） | Wuhan | 81 | -70（246/316） |
| 10 | Qingdao | 6 | -8（3/11） | Wuxi | 14 | -19（16/35） | Jinan | 34 | -2（74/76） | Qingdao | 79 | 138（315/177） |
| 10 | Tianjin | 6 | -9（10/19） |  |  |  | Qingdao | 34 | 39（70/31） |  |  |  |
| 10 | Wuhan | 6 | 8（10/2） |  |  |  | Zhengzhou | 34 | -121（191/40） |  |  |  |
| 10 | Xian | 6 | -5（3/8） |  |  |  |  |  |  |  |  |  |

Note: The figures in parentheses represent the weighted in-degree and weighted out-degree of the city, respectively.

NID is an abbreviation for Net Inflow Degree.

**Appendix Table 2** Top 10 Cities by Centrality in the Innovation Network from a Local Perspective

| Rank | **Phase I** | | | **Phase II** | | | **Phase III** | | | **Phase IV** | | |
| --- | --- | --- | --- | --- | --- | --- | --- | --- | --- | --- | --- | --- |
|  | City | Centrality | NID | City | Centrality | NID | City | Centrality | NID | City | Centrality | NID |
| 1 | Shanghai | 12 | -15（6/21） | Shanghai | 19 | -42（54/96） | Shanghai | 38 | -19（164/183） | Shanghai | 51 | -138（513/651） |
| 2 | Nanjing | 7 | -3（3/6） | Nanjing | 16 | -33（30/63） | Nanjing | 27 | 78（214/136） | Nanjing | 42 | 240（424/184） |
| 3 | Suzhou | 6 | 8（13/5） | Hangzhou | 11 | -21（30/51） | Hangzhou | 20 | -6（57/63） | Hangzhou | 40 | 48（240/192） |
| 4 | Hangzhou | 3 | -1（3/4） | Wuxi | 11 | -6（14/20） | Wuxi | 20 | 3（34/31） | Suzhou | 33 | 83（430/347） |
| 5 | Wuxi | 3 | -1（4/5） | Changzhou | 7 | -14（21/35） | Suzhou | 19 | -54（129/183） | Hefei | 28 | 4（53/49） |
| 6 | Hefei | 2 | -6（0/6） | Ningbo | 6 | -1（3/4） | Changzhou | 12 | 10（37/27） | Wuxi | 23 | 8（84/76） |
| 7 | Nantong | 2 | 4（4/0） | Suzhou | 6 | 51（64/13） | Hefei | 11 | 4（19/15） | Ningbo | 21 | 14（103/89） |
| 8 | Ningbo | 2 | 5（5/0） | Zhenjiang | 6 | 28（33/5） | Ningbo | 10 | 18（26/8） | Nantong | 17 | 102（162/60） |
| 9 | Taizhou | 2 | 1（2/1） | Nantong | 4 | 4（6/2） | Nantong | 9 | 3（26/23） | Yangzhou | 17 | 14（39/25） |
| 10 | Zhenjiang | 2 | -2（0/2） | Jiaxing | 3 | 3（6/3） | Wenzhou | 9 | -5（6/11） | Jiaxing | 16 | 29（128/99） |
| 10 |  |  |  | Taizhou | 3 | -1（2/3） |  |  |  |  |  |  |
| 10 |  |  |  | Yancheng | 3 | 4（6/2） |  |  |  |  |  |  |
| 10 |  |  |  | Yangzhou | 3 | 6（7/1） |  |  |  |  |  |  |

Note: NID is an abbreviation for Net Inflow Degree.

**Appendix Table 3** Results of the analysis of influencing factors

| **Impact Dimension** | **Influencing Factor** | **Indicator Name** | **National Perspective** | | **Local Perspective** | |
| --- | --- | --- | --- | --- | --- | --- |
|  |  |  | **Correlation** | **Significance** | **Correlation** | **Significance** |
| **Economic Development Dimension** | Economic Development Level | Regional Gross Domestic Product (GDP) | 0.794 | 0.000 | 0.748 | 0.000 |
|  | Financial Support Strength | Balance of RMB Loans in Financial Institutions at Year-End | 0.819 | 0.000 | 0.815 | 0.000 |
|  | Consumer Market Activity | Total Retail Sales of Consumer Goods | 0.742 | 0.000 | 0.809 | 0.000 |
|  | Industrial Economy Scale | Operating Revenue of Industrial Enterprises above Designated Size | 0.738 | 0.000 | 0.713 | 0.000 |
|  | Trade Openness | Total Import and Export Value of Goods | 0.802 | 0.000 | 0.629 | 0.032 |
|  | Modernization Level of Economic Structure | Share of Secondary and Tertiary Industries in GDP | 0.177 | 0.000 | 0.406 | 0.005 |
| **Technological Innovation Dimension** | Marketization Capability of Technological Achievements | Turnover of Technology Contracts | 0.661 | 0.000 | 0.779 | 0.000 |
|  | Output Level of Technological Innovation | Number of Granted Patents | 0.838 | 0.000 | 0.692 | 0.003 |
|  | Density of Core Innovation Entities in Cities | Number of National High-Tech Enterprises | 0.559 | 0.000 | 0.387 | 0.076 |
| **Policy/Social Dimension** | Talent Development Support | Education Expenditure | 0.726 | 0.000 | 0.672 | 0.005 |
|  | Technological Innovation Support | Science and Technology Expenditure | 0.811 | 0.000 | 0.677 | 0.004 |
|  | Higher Education Resources | Number of Higher Education Institutions | 0.512 | 0.000 | 0.756 | 0.000 |
|  | Development Zone Policies | Number of Development Zones | 0.552 | 0.000 | 0.809 | 0.000 |
| **Infrastructure Dimension** | Level of Communication Infrastructure | Revenue from Postal and Telecommunication Services | 0.725 | 0.000 | 0.613 | 0.016 |
|  | Level of Medical Resources | Number of Hospital Beds | 0.668 | 0.003 | 0.623 | 0.000 |
